# Improved Homologous Expression of the Acidic Lipase from *Aspergillus niger*

**DOI:** 10.4014/jmb.1906.06028

**Published:** 2019-11-22

**Authors:** Si-Yuan Zhu, Yan Xu, Xiao-Wei Yu

**Affiliations:** Key Laboratory of Industrial Biotechnology, Ministry of Education, School of Biotechnology, Jiangnan University, Wuxi 214122, P.R. China

**Keywords:** *Aspergillus niger*, acidic lipase, expression, characterization, *Agrobacterium-*mediated transformation

## Abstract

In this study, the acidic lipase from *Aspergillus niger* (*ANL*) was homologously expressed in *A. niger*. The expression of ANL was significantly improved by the expression of the native ANL with the introns, the addition of the Kozak sequence and the optimization of the signal sequences. When the cDNA sequence of *ANL* fused with the *glaA* signal was expressed under the *gpdA* promoter in *A. niger*, no lipase activity could be detected. We then tried to improve the expression by using the full-length *ANL* gene containing three introns, and the lipase activity in the supernatant reached 75.80 U/ml, probably as a result of a more stable mRNA structure. The expression was further improved to 100.60 U/ml by introducing a Kozak sequence around the start codon due to a higher translation efficiency. Finally, the effects of three signal sequences including the *cbhI* signal, the *ANL* signal and the *glaA* signal on the lipase expression were evaluated. The transformant with the *cbhI* signal showed the highest lipase activity (314.67 U/ml), which was 1.90-fold and 3.13-fold higher than those with the *ANL* signal and the *glaA* signal, respectively. The acidic lipase was characterized and its highest activity was detected at pH 3.0 and a temperature of 45ºC. These results provided promising strategies for the production of the acidic lipase from *A. niger*.

## 1. Introduction

*Aspergillus niger* is one of the most important microorganisms in the production of organic acids and a wide range of enzymes [1]. Many substances produced in *A. niger* are considered as Generally Recognized as Safe (GRAS) by the United States Food and Drug Administration [1]. A variety of industrially important enzymes such as oxidases, cellulases, dehydrogenases, hydrolases and pectinases have been produced in *A. niger* [2]. In addition, other structurally complex antibody proteins or small peptides, such as nucleoside hydrolase, sterol 24-c-methyltransferase, humanized immunoglobulin G1 antibodies, cyclodepsi-peptides (octa-enniatin and octa-beauvericin), were also successfully expressed in *A. niger* [3-6]. In recent years, *A. niger* has been used for the production of high-value recombinant antibodies with a lower cost since the protein produced in *A. niger* is processed more similarly to mammals [6, 7]. The products from *A. niger* have been widely applied in food, feed, bioenergy and pharmaceutical processes, amongst others [2].

Many attempts have been made to increase the expression of proteins in filamentous fungi [8], for example, by optimi-zation of promoters and copy numbers, or expression as fusion protein [9-11]. The expression of xylanase was improved by 2.27-fold through modification of the *gpdA* promoter [12]. The *A. niger* glucoamylase gene is commonly used as a fusion tag for improvement of foreign gene expression in *A. niger*, including the porcine pancreatic prophospholipase A2 (proPLA2) gene [11], the human lymphotoxin alpha (*LTα*) gene [9] and the catalytic subunit of the bovine enterokinase (*EKL*) gene [10].

In addition, a superior host strain is very important to in achieving target protein expression at a high level. A host with a lower protein background could be obtained by disrupting the gene of the original high-yield protein in the strain. Without the interference of endogenous proteins, a higher yield could be obtained for recombinant proteins in the host. At present, tannase, xylanase, mannase and asparaginase have been successfully expressed at a high level in strains with a lower endogenous protein background [13, 14]. In another example, Kamaruddin *et al*. [15] increased the yield of cutinase by 36-fold in *A. niger* by down-regulating the protease expression.

Acidic lipases are in a category of extreme enzymes that are stable and exhibit high catalytic activity in extreme acidic conditions. Due to the acidogenic properties of *A. niger*, most of the proteins or metabolites in *A. niger* are acid-tolerant. The *A. niger* lipase (*ANL*) is an acidic lipase that maintains high activity under acidic conditions and has a wide range of applications in food and chemical industries [16]. ANL has exhibited better resistance to acidic conditions compared to the other *Aspergillus* sp. lipases [17, 18]. For example, the lipase from A. carneus was not stable at pH 6 as its optimum pH was 9.0 [17], and the highest stability of the lipase from *A. awamori* was at pH 8.0-9.0 [18]. However, the high cost for the production of the acidic *A. niger* lipase remains a barrier for its industrial applications. At present, ANL has been heterologously expressed in *Escherichia coli* and *Pichia pastoris*. The specific activity of the ANL was 110 U/(mg protein) in *E. coli* and could be improved two folds by addition of Ca^2+^ [19]. When expressed in *P. patoris* the activity of ANL fused with a small ubiquitin-related modifier (SUMO) reached 173 U/(ml supernatant) and 432 U/(mg protein) in the shake flasks [20].

In this study, the *ANL* gene was homologously expressed in *A. niger*. Improvement of the *A. niger* lipase (*ANL*) expression was achieved through three strategies, including the addition of Kozak sequences for enhancing the translation efficiency, the expression of the native ANL with the introns for improving the stability of the mRNA, and optimization of signal sequences for enhancing the efficiency of extracellular secretion.

## Materials and Methods

### Strains, Plasmids, Media and Culture Conditions

*E. coli* TOP10 was used to maintain and amplify the plasmids. *A. niger* 89 was preserved in our lab and used as the expression host, as it has a low-background of endogenous secretory proteins and is hygromycin B-sensitive. *Agrobacterium tumefaciens* AGL1 was used for *A. tumefaciens*-mediated transformation (ATMT).

LB medium (1% w/v peptone, 0.5% w/v yeast extract, 1% w/v NaCl with 50 μg/ml kanamycin) was used for cultivation of *E. coli*. YEB medium (0.1% w/v yeast extract, 0.5% w/v peptone, 0.5% w/v beef extract, 0.5% w/v sucrose, 0.0493% w/v MgSO_4_·7H_2_O, pH 7.0 with 50 μg/ml Rif ) was used for the *A. tumefaciens* growth at 28°C 200 rpm for 12-16 h. IM medium (0.145% w/v KH_2_PO_4_, 0.205% w/v K_2_HPO_4_, 0.05% w/v (NH_4_)_2_SO_4_, 0.05% w/v MgSO_4_·7H_2_O, 0.015% w/v NaCl, 0.0066% w/v CaCl_2_, 0.000248% w/v FeSO_4_·7H_2_O, 0.18% w/v glucose, 0.5% v/v glycerin with 40 mM MES (pH5.3) and 200 μM Acetosyringone (AS)) was used for pre-incubation of *A. tumefaciens* at 28ºC 200 rpm and IM medium supplemented with 2% agar (IM plate) was used for co-cultivation of *A. tumefaciens* and *A. niger* at 23ºC. The potato dextrose agar (PDA plate, OKA Co. Ltd., China) was used for sporulation of *A. niger*. Fermentation medium (0.2% w/v NaNO_3_, 0.05% w/v KCl, 0.05% w/v MgSO_4_·7H_2_O, 0.1% w/v K_2_HPO_4_·3H_2_O, 0.001% w/v FeSO_4_·7H_2_O, 1% w/v (NH_4_)_2_SO_4_, 4% w/v corn starch, 2% v/v olive oil, 10% v/v 10 × 100 mmol/l pH 6.0 phosphate buffer) was used for the protein expression in *A. niger*. The rhodamine-olive oil plate (0.2% w/v NaNO_3_, 0.05% w/v KCl, 0.05% w/v MgSO_4_·7H_2_O, 0.1% w/v K_2_HPO_4_·3H_2_O, 0.25% w/v glucose, 0.25% w/v corn starch, 0.25% w/v maltose, 10% v/v 10 × 100 mmol/l pH 6.0 phosphate buffer, 1*10-3% w/v Rhodamine B (Biosharp Co. Ltd., China), 1% v/v olive oil emulsion (olive oil was emulsified with 4% w/v polyvinyl alcohol (PVA) in a ratio of 1:3 (v/v) by a high-speed homogenizer), 2% w/v agar) was used for screening.

### Construction of Recombinant Strains

**Fig. 1:**
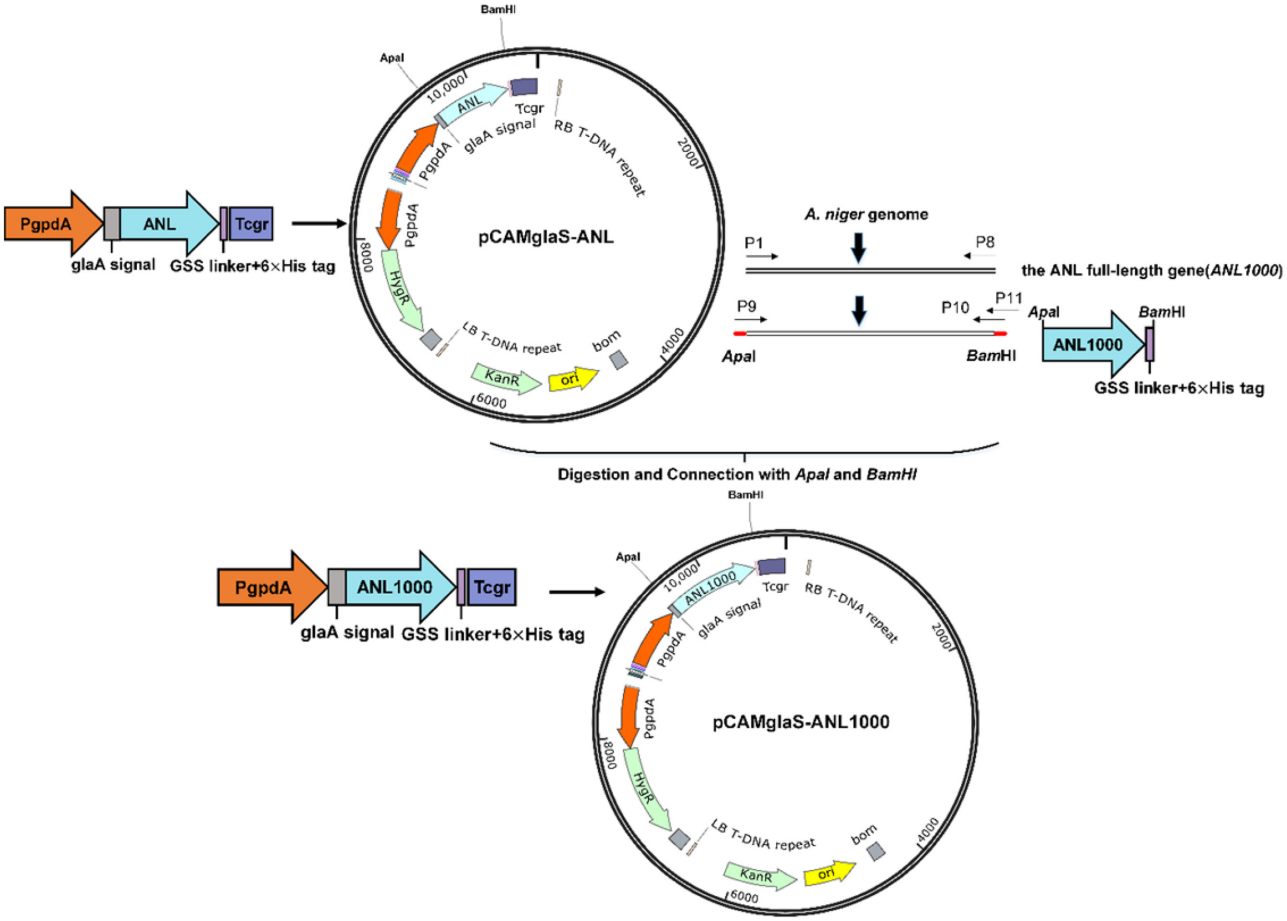
Construction of pCAMglaS-ANL and pCAMglaS-ANL1000 expression vectors.

**Fig. 2:**
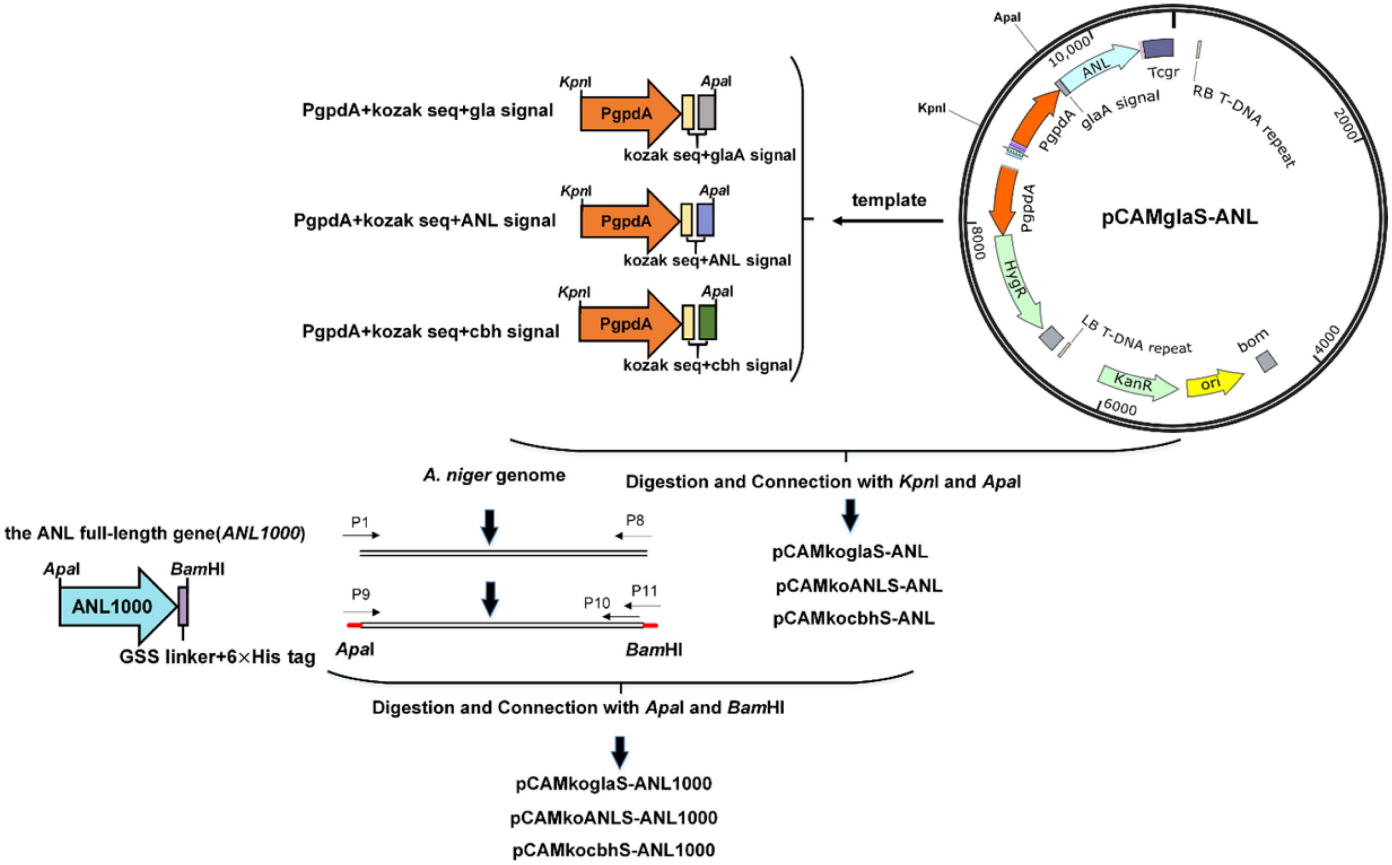
Construction of 6 kinds of expression vectors.

The full-length gene of *ANL* (*ANL1000*) (GenBank: DQ647700.1) contains three introns. *ANL1000* was amplified from the *A. niger* genome and the intron-free *ANL* gene (*ANL*) was amplified by overlap extension PCR. In the plasmid pCAMglaS-ANL, the intron-free *ANL* gene was fused with the *glaA* signal sequence and expressed under the *gpdA* promoter. The *ANL* gene in pCAMglaSANL was replaced with *ANL1000* by restriction enzyme digestion (ApaI and BamHI) to construct the plasmid pCAMglaS-*ANL1000* (Fig. 1). Three kinds of signal sequences were used, those of glucoamylase, ANL and exoglucanase cbhI. The signal sequences were introduced through PCR. Six vectors were constructed with different combinations of the three signal sequences and two lengths of *A. niger* lipase gene (*ANL*, *ANL1000*). According to the design principle of the kozak sequence [21], the kozak sequence (GCCA-3CCA^+1^TGG^+4^) was added around the start codon A^+1^TG, and two other nucleotides were added downstream of the Kozak sequence to avoid frameshift. As indicated in Table S3, the other two added nucleotides are AT for the *glaA* signal and AG for the *ANL* signal and the *cbhI* signal right after the G^+4^. All plasmids contained the Hyg marker gene for selection on hygromycin B. 6×his-tag was added at the C-terminus of the lipase for purification of the expressed enzyme by affinity chromatography. The work flow for construction of the plasmids is shown in Fig. 2. All primers used are listed in Table S1. All vectors proved to be correctly assembled by restriction endonuclease digestion and sequencing of the assembly joint.

### *Agrobacterium-*Mediated Transformation (ATMT)

In the recombinant plasmid the fragment including the target gene between two elements, RB (right boundary) and LB (left boundary), was randomly inserted into the genome of *A. niger* by ATMT. The recombinant plasmids were transformed into *A. tumefaciens* AGL1 by the freeze-thaw method [22]. Positive transformants of AGL1 were cultivated in 5 mL of YEB at 28ºC for 16 h, and then collected and incubated in IM liquid medium at 28ºC to an OD_600_ of 0.8-1.0. *A. niger* spore suspension was generated from strains cultivated for 3-5 days at 30ºC. A mixture of 100 μl of the *A. niger* spore suspension (107 spores/ml) and 100 μl of the positive transformant of AGL1 was spread on the cellophane covering the IM plates. The co-cultivation was carried out in the dark at 23ºC for 48 h. Afterwards, the cellophane was transferred to the PDA plates with hygromycin B (200 μg/ml) and cefotaxime sodium (200 μg/ml). The plates were incubated at 30ºC for 2-3 d. Then, the transformants were rescreened on the PDA plates with hygromycin B (200 μg/ml). The transformants were purified by spore isolation for at least three successive generations on the PDA plates with hygromycin B (200 μg/ml). The positive transformants were confirmed by PCR with the identification primers (Table S2).

### Expression of ANL in *A. niger*

The wild-type strain and the positive transformants of *A. niger* were grown on the PDA plates for 3-5 d, and then the spores were washed with saline. The spore suspension was inoculated into 50 ml fermentation medium with a final concentration of 2 × 10^5^ spores/ml in 250 ml flasks shaken (200 rpm) at 28ºC for 168 h. Protein concentrations of the supernatant were determined by the Bradford method [23].

### Purification and Identification of ANL

ANL fused with a 6×his-tag at the C-terminus was purified by Ni-NTA column affinity chromatography using the ÄKTA protein purification system. The fermentation supernatants were collected by filtering through a 200-mesh nylon membrane, then filtered through 0.22 μm aqueous microfiltration membrane. The system was equilibrated with buffer A (150 mM NaCl, 20 mM Tris-HCl, pH 8.0) and the target protein was eluted with buffer B (150 mM NaCl, 20 mM Tris-HCl, 0.5 M imidazole, pH 8.0). The purified protein was examined by SDS-PAGE and stored in buffer A at -80ºC for further analysis. For preparation of the SDS-PAGE samples, the purified protein solution or the fermentation supernatant was mixed with the 2X SDS loading buffer in equal volume. The samples were boiled for 10 min, centrifuged for 5 min, and then 10 μl of each sample was loaded into the SDS-PAGE gel.

Matrix-Assisted Laser Desorption/Ionization Time-of-Flight Mass Spectrometry (MALDI-TOF-MS) was used for protein identification. The target protein on SDS-PAGE was cut out and digested. The peptide mass fingerprinting of the target protein was analyzed using Mascot software (http://www.matrixscience.com).

### Assay of Enzyme Activity

The enzyme activity of ANL was determined by alkali titration using olive oil as the substrate. Olive oil was emulsified with 4%w/v polyvinyl alcohol (PVA) at a ratio of 1:3 (v/v) by a high-speed homogenizer and used as the substrate. Each reaction contained 4 ml of substrate and 5 ml of 50 mM citrate-phosphate buffer (pH 3.0) and 1 ml enzyme solution. For measurement of the purified enzyme, the activity assays were done using 1 ml of 0.25 mg /ml enzyme solution. For measurement of the activity of the supernatant, the activity assays were done using 1 ml of the supernatant. The reaction was incubated at 45ºC for 15 min, and then terminated by adding 15 ml of 95% ethanol solution. The heat-inactivated enzyme solution was used as the blank control. The reaction solution was titrated with 0.1 mol/l NaOH standard solution. The amount of enzyme that produces 1 μmol of fatty acid in one minute is defined as one unit of enzyme activity. The enzyme activity was calculated using the following equation.

$$X=\frac{(B-A)\mathrm{\times C\times n}}{t}$$

X: enzyme activity, U/L; B: volume of NaOH consumed by titrating the sample, ml; A: volume of NaOH consumed by titrating the blank; C: NaOH concentration, mol/L; n: dilution factor; t: reaction time.

### Characterization of the Purified Enzyme ANL

The optimum pH of the purified enzyme ANL was determined by measuring the lipase activity at 45ºC in various 50 mM citrate-phosphate buffers (pH 2.0-9.0). The pH stability was studied by incubating the purified enzyme ANL in different 50 mM citrate-phosphate buffers with pH ranging from 2.0 to 10.0 for 24 h at 45ºC. The samples were taken at 2-h intervals for the first 12 h and for the last timepoint at 24 h. The residual activity was then assayed under the standard assay conditions.

The optimum temperature of the purified enzyme ANL was determined by measuring the enzyme activity at various temperatures (25ºC-60ºC) in 50 mM of citrate-phosphate buffer, pH 3. The thermal stability was determined by incubating the purified enzyme ANL for 24 h at the desired temperatures (30ºC-60ºC) followed by measuring the residual activity. The samples were taken at 2-h intervals for the first 12 h and for the last timepoint at 24 h.

The kinetic parameters of ANL were determined using the emulsified triglyceride olive oil (0-120 g/l) as the substrate and assayed using the alkali titration method under optimum assay conditions. The values of the kinetic parameters *K*_m_ and *k*_cat_ were determined by Lineweaver-Burk plot and Michaelis-Menten equation.

## Results and Discussion

### Design of the Expression Vectors

The expression cassettes of the plasmids are shown in Table 1. ANL is the cDNA gene without intron, and *ANL1000* represents the native full-length *ANL* gene containing three introns. The length of the native *ANL* gene is about 1,000 bp. Thus, we named the native ANL ‘*ANL1000*.’ The Kozak sequence (GCCA^-3^CCA+1TGG^+4^) was added around the start codon of the *ANL* gene in the plasmid pCAMkoglaS-ANL, and two other nucleotides were added downstream of the kozak sequence G^+4^ to form a codon to avoid frameshift. Three signal sequences were selected including the glucoamylase (*glaA*) signal sequence, the *ANL* signal sequence and the cellobiohydrolase I (*cbhI*) signal sequence for the expression of ANL in *A. niger*. The *glaA* signal sequence is used widely in *A. niger*, because glucoamylase is expressed in high amounts in *A. niger* [24], and the signal sequence from a highly expressed gene usually helps the extracellular expression of the target gene [25]. The *ANL* signal sequence is its own signal sequence. Zoglowek *et al*.[8] suggest that natural signal sequence may be more suitable for gene expression than other signal sequences. The *cbhI* signal sequence was also used frequently in *A. niger*. In the study of Madhavan *et al*. [26], the *cbhI* signal sequence exhibited higher secretion than the *glaA* signal sequence. The signal sequences are listed in Table S3.

### Expression of ANL in *A. niger*

**Fig. 3:**
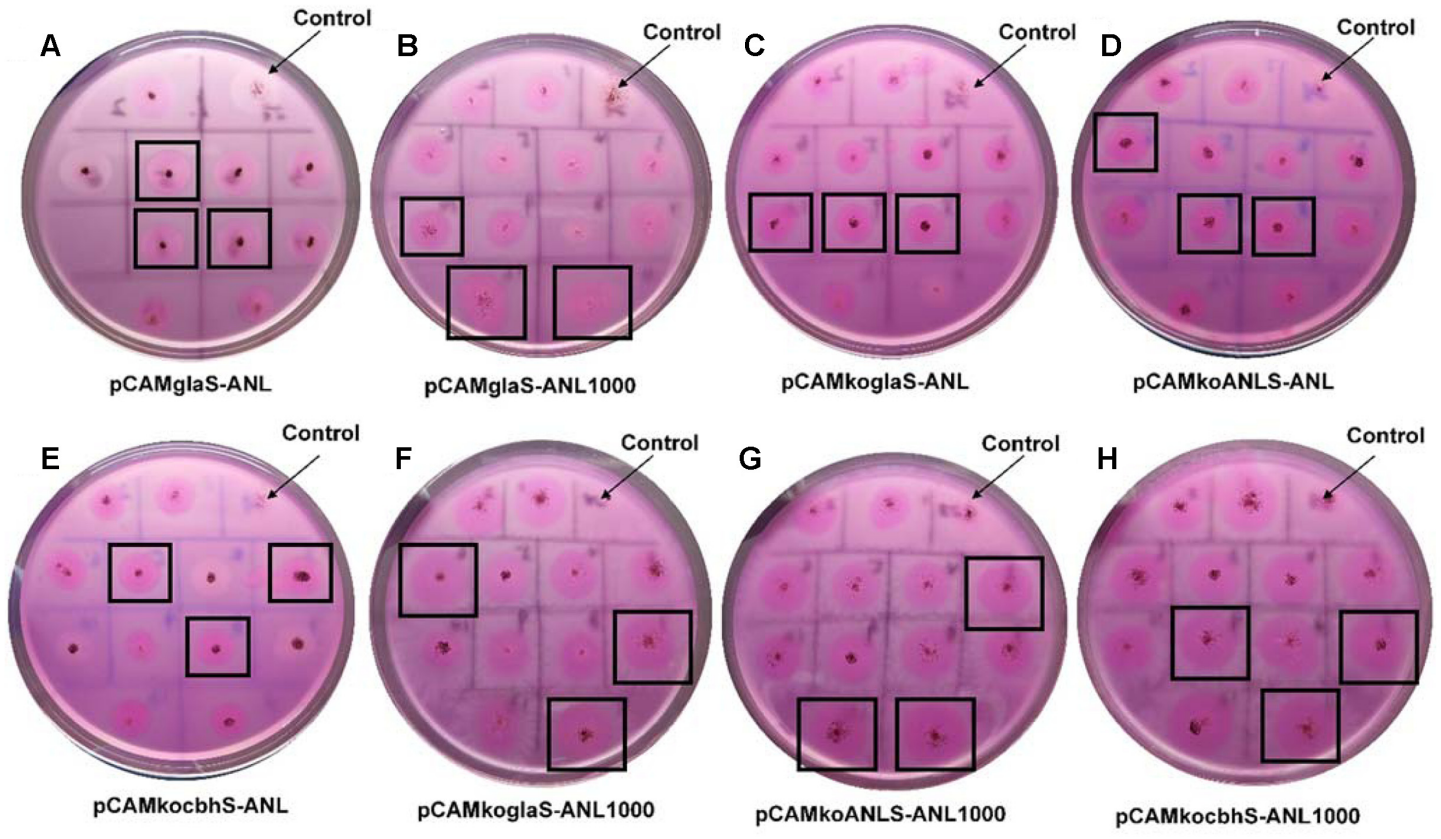
*A. niger* 89 transformed with eight different plasmids grown on the rhodamine-olive oil plates for 2 days (the wild-type *A. niger* 89 as the control). The letters represent 8 different constructs. The bigger the red halo, the higher the lipase activity. To determine the lipase activity shown in Fig. 4 we selected three transformants for each construct with relatively bigger halos indicated with a square.

**Identification of positive transformants.** We isolated several *A. niger* strains from the environment. The endogenous secretory proteins in the supernatant were detected on SDS-PAGE and we found that the secretory proteins in *A. niger* 89 were much lower than those in other strains (data not shown). Thus, we chose *A. niger* 89 as the expression host in our study. The plasmids indicated in Table 1 were transformed into *A. niger* 89 by ATMT for the expression of ANL. The positive transformants verified by PCR were plated on the rhodamine-olive oil plate as shown in Fig. 3. The lipase produced by the transformant can hydrolyze olive oil to generate fatty acids. The fatty acids then react with rhodamine B to develop a red color under natural light and to emit fluorescence under ultraviolet light [27]. The larger the color halo around the colony, the higher the lipase activity. Thus, the positive transformants with higher lipase activity were selected according to the size of the color halo. The wild-type *A. niger* 89 didn’t show an obvious color halo. The other 8 kinds of transformants showed different sizes of color halos on the rhodamine-olive oil plates. Although transformed with the same plasmid, the expression level of lipase varied among different transformants due to the random insertion of the expression cassette in the genome [26].

**Fig. 4:**
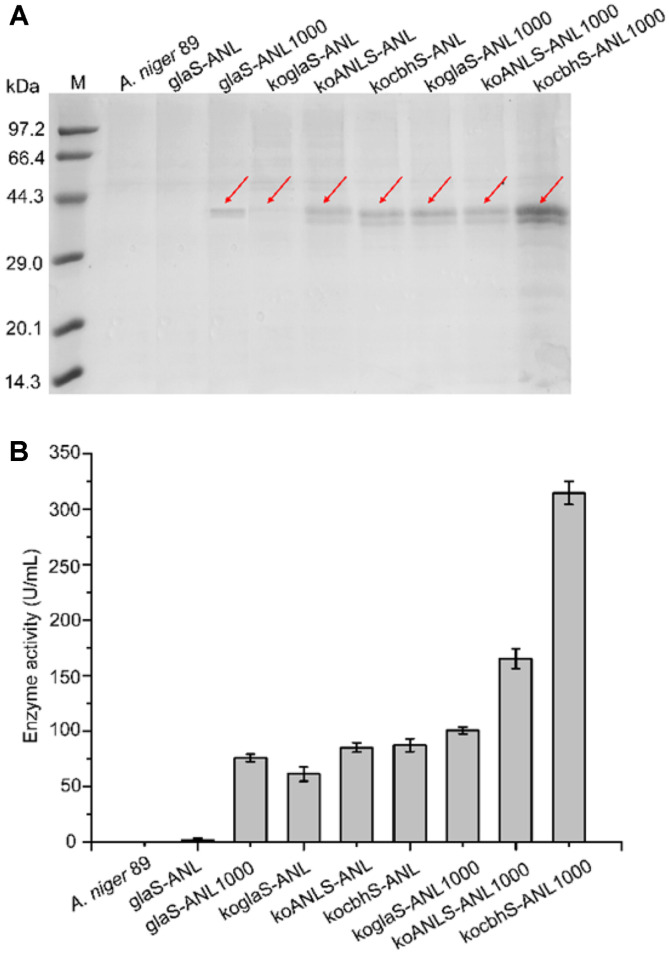
SDS-PAGE and the lipase activities of the transformants. (**A**) SDS-PAGE of the fermentation supernatants after cultivation for 168 h. (**B**) Enzyme production in the supernatants after cultivation for 168 h. The lipase activity was measured on emulsified olive oil at pH 3.0 and 45ºC. Error bars indicated the standard deviation of the enzyme activities of three transformants for each construct in biological triplicates.

**The effects of the introns, the Kozak sequence and the signal sequences on the ANL expression.** The wild-type strain *A. niger* 89 and the transformants were cultivated using corn starch and olive oil as the main carbon sources. Olive oil was used both for the carbon source and the inducer of the lipase. We followed the lipase production during fermentation. The lipase began to secrete into the medium between 48 h and 72 h and the highest lipase production was reached at around 168 h during the fermentation. The wild-type strain *A. niger* 89, the transformants of pCAMglaS-ANL and pCAMglaS-*ANL1000* were characterized for the effect of the introns. As shown in Fig. 4A, there was no obvious target band for either the wild type (*A. niger* 89) or the transformants of pCAMglaS-ANL (glaS-ANL). The target band for the transformant of pCAMglaS-*ANL1000* with the introns (glaS-*ANL1000*) could be detected and the lipase activity in the supernatant reached 75.80 U/ml. Although introns do not participate in protein-coding sequences, they do play significant functional roles in eukaryotes, including regulation of alternative splicing, positive regulation of gene expression, regulation of nonsense-mediated decay, and provision of a new gene source as well as impact on natural selection [28]. In our case, the presence of the introns may enable a more stable secondary structure, which protects pre-mRNA from degradation in the nucleus [29, 30]. Moreover, the introns can assist in the transport of pre-mRNA [31] and regulation of mRNA maturation [32]. In this study, the expression of the *ANL* gene with introns was significantly higher compared to the cDNA.

The effect of the Kozak sequence on the lipase expression was also evaluated using two sets of comparison (pCAMglaS-ANL vs pCAMkoglaS-ANL, pCAMglaS-*ANL1000* vs pCAMkoglaS-*ANL1000*). After addition of the kozak sequence in pCAMkoglaS-ANL, the lipase activity increased from undetectable to 61.30 U/ml. When the Kozak sequence was added in pCAMkoglaS-*ANL1000* with the introns, the lipase activity was improved by 1.33-fold compared with that of pCAMglaS-*ANL1000*.

Kozak *et al*. [21] found that the base pair near the initiation codon has a certain effect in translation, and the certain sequence, GCCACCATGG, is known as the ‘Kozak sequence’ and shows a higher translation level. The Kozak sequence enhances the translation efficiency by optimization of the ATG environment to avoid the leaky ribosomal-scanning [33]. The Kozak sequence has been applied to increase the expression level of foreign genes in mammalian cells [34, 35] and *Saccharomyces cerevisiae* [36], but there is no report on the effect of the Kozak sequence on protein expression in *A. niger*.

The effects of three signal sequences (*glaA* signal sequence, *ANL* signal sequence and *cbhI* signal sequence) were analyzed and tested with both ANL1000 and *ANL*. As shown in Fig. 4 and Table 2, the enzyme production and protein concentration in the supernatant of the transformants were evaluated. The enzyme production in the supernatant has a positive correlation with the protein concentration. Higher enzyme activity per milliliter (U/ml) indicates higher enzyme production in the supernatant. The enzyme production of the pCAMkocbhS-ANL1000 transformant (kocbhS-ANL1000) was the highest (314.67 U/ml), followed by koANLS-ANL1000, koglaS-ANL1000, kocbhS-ANL, koANLS-ANL, glaS-ANL1000, koglaS-ANL, glaS-ANL. The enzyme production of all ANL1000 proteins was higher compared to ANL. Regarding the signal sequence, the transformants with the *cbhI* signal sequence showed the highest expression, followed by those with the ANL and *glaA* signal sequence. It is worth noting that the thickness of the protein bands on SDS-PAGE was not correlated very well with the average protein concentration in Table 2. The reason was that the loading sample for each construct was not a mixture of three transformants, but one randomly selected from these replicates.

### Purification and Identification of ANL

**Fig. 5:**
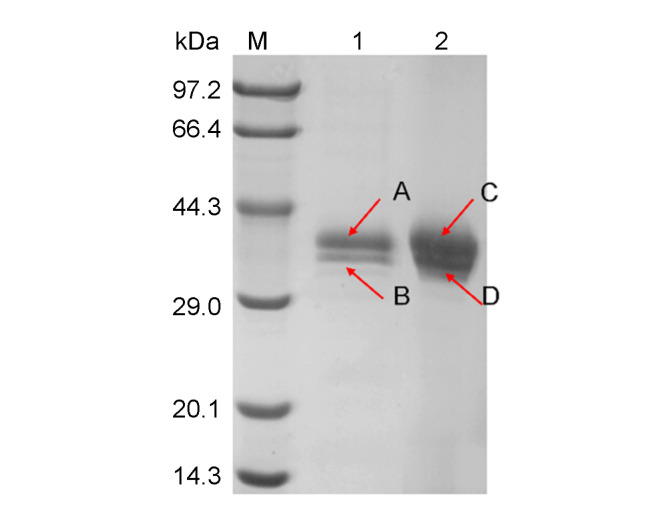
SDS-PAGE of the fermentation supernatant and the purified protein. 1. The fermentation supernatant of kocbhS-*ANL1000* after cultivation for 168 h. 2. The purified protein loaded with 3 μg. Band A, B, C, and D were cut from gel and analyzed using MALDI-TOF-MS.

As shown in Fig. 5 the fermentation supernatant and the purified enzyme were detected by SDS-PAGE. About 0.15 g of ANL enzyme was purified from 1 L fermentation supernatant. The specific activity of the purified ANL is 680.34 ± 4.75 U/mg. Two bands were detected for both the fermentation supernatant samples and the purified protein. The four bands of proteins were identified by MALDI-TOF-MS. The results showed that band-A and band-C contained four unique fragments of VTHLNDIVPR, VGNYALAEHITSQGSGANFR, MLLEFDLTNNFGGTAGF LAADNTNKR and NDGYSVELYTYGCPR, the band-B and the band-D contained two unique fragments of VGNYALAEHITSQGSGANFR and VTHLNDIVPR. By comparison, all these unique fragments belong to ANL. The difference in size may be due to post-translational modifications such as glycosylation.

### Enzymatic Characterization 

**Fig. 6:**
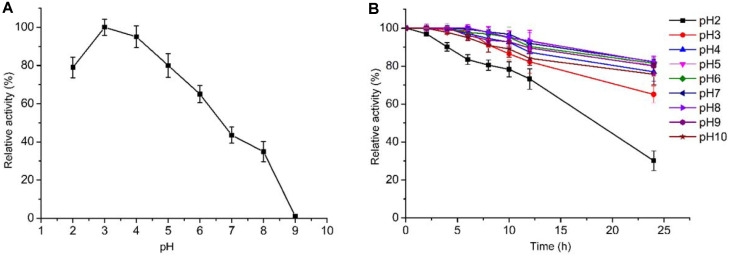
Effect of pH on the activity and stability of ANL. (**A**) The optimal pH. The activity was determined at pH 2.0-9.0 and 45ºC using olive oil as the substrate. (**B**) The pH stability of ANL. The pH stability of ANL was determined at 45ºC and pH 3.0 after being incubated in pH 2.0-10.0 50 mM citrate-phosphate buffer for up to 24 h. Error bars indicate the standard deviation of three biological replicates.

**Effects of pH on the lipase activity and stability.** As shown in Fig. 6A the optimum pH of ANL is pH 3. ANL exhibits a high stability in a broad range of pH from 3 to 10. After being retained under pH 3 for 24 h, the relative activity of ANL is still more than 60% (Fig. 6B). In other reports the optimum pH of ANL was around 2.5-5 [37-39]. During fermentation *A. niger* produces a high amount of acid, which might be the reason for a very acid-resistant ANL.

**Fig. 7:**
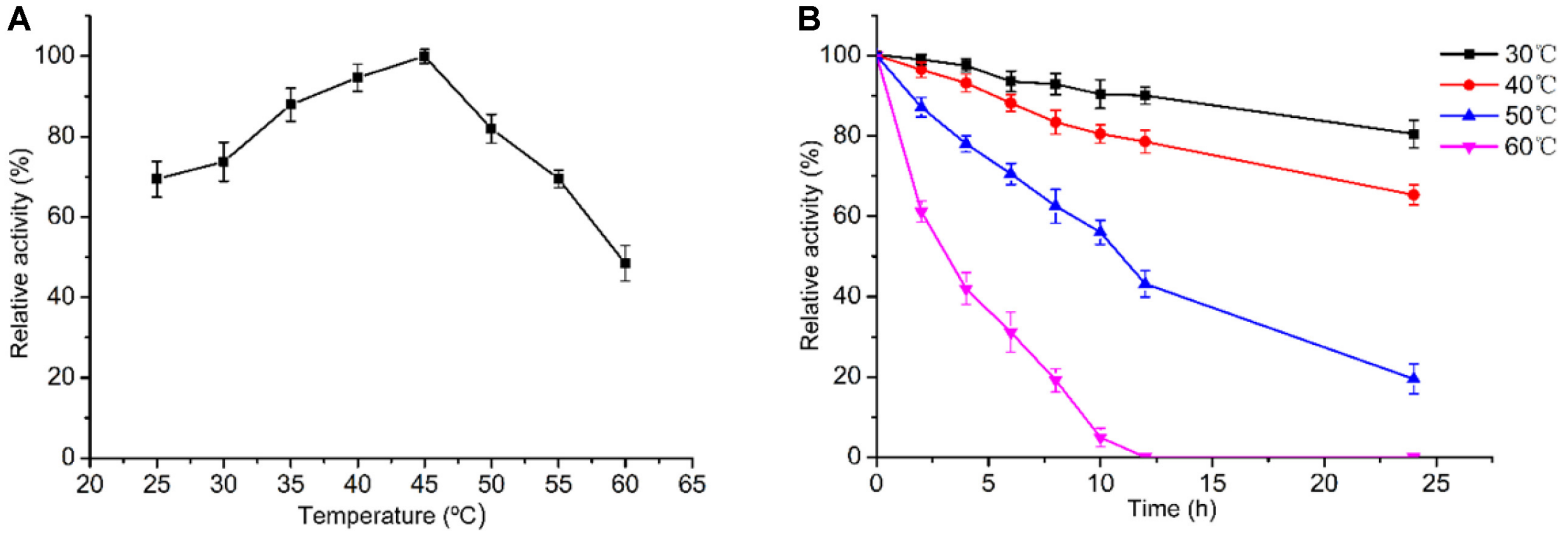
Effect of temperature on the activity and stability of ANL. (**A**) The optimal temperature. The activity was determined at 25-60ºC and pH 3.0. (**B**) The temperature stability of ANL. The temperature stability of ANL was determined at 45ºC and pH 3.0 after incubation for up to 24 h at temperatures of 30-60ºC. Error bars indicate the standard deviation of three biological replicates.

**Effects of temperature on the lipase activity and stability.** As shown in Fig. 7 the optimum temperature of ANL is 45ºC. In the analysis of thermostability, the relative activity of the purified ANL was still more than 70% after incubation at 30ºC and 40ºC for 24 h. However, the relative activity decreased significantly when incubated at 50ºC for 12 h. At 60ºC the relative activity dropped to 30% within 5 h and was completely inactivated within 12 h.

**Determination of kinetic parameters.** The olive oil emulsion was used as the substrate to determine the kinetic parameters. As shown in Table 3, the *K*_m_ and *k*_cat_ values are 9.30 ± 1.04 g/l and 391.66 ± 8.69 s^-1^, respectively. *K*_m_ reflects the affinity of the enzyme to the substrate. The lower the *K*_m_, the higher the affinity with the substrate. In this study, ANL has a better affinity towards olive oil compared with other studies in which the Km values of the lipases from *A. niger* were 77 mM (22 g/l) [39] and 108 g/l [16] using olive oil as the substrates.

## Supplemental Materials



Supplementary data for this paper are available on-line only at http://jmb.or.kr.

## Figures and Tables

**Table 1:** Plasmids used for the acidic lipase expression.

	Plasmid	Transformants	Expression cassette
1	pCAMglaS-ANL	glaS-ANL	PgpdA- *glaA* signal-ANL-his tag-Tcgr
2	pCAMglaS-ANL1000	glaS-ANL1000	PgpdA- *glaA* signal-ANL1000- his tag-Tcgr
3	pCAMkoglaS-ANL	koglaS-ANL	PgpdA-kozak seq-*glaA* signal-ANL- his tag-Tcgr
4	pCAMkoANLS-ANL	koANLS-ANL	PgpdA-kozak seq-ANL signal-ANL- his tag-Tcgr
5	pCAMkocbhS-ANL	kocbhS-ANL	PgpdA-kozak seq-cbhⅠ signal-ANL- his tag-Tcgr
6	pCAMkoglaS-ANL1000	koglaS-ANL1000	PgpdA-kozak seq-*glaA* signal-ANL1000- his tag-Tcgr
7	pCAMkoANLS-ANL1000	koANLS-ANL1000	PgpdA-kozak seq-ANL signal-ANL1000- his tag-Tcgr
8	pCAMkocbhS-ANL1000	kocbhS-ANL1000	PgpdA-kozak seq-cbhⅠ signal-ANL1000- his tag-Tcgr

**Table 2:** Protein concentration in the supernatant of the transformants after cultivation for 168 h.

Transformants	Protein concentration (mg/mL)^*^
*A. niger* 89	0.08±0.01
glaS-ANL	0.09±0.01
glaS-*ANL1000*	0.14±0.02
koglaS-ANL	0.12±0.01
koANLS-ANL	0.18±0.02
kocbhS-ANL	0.19±0.02
koglaS-*ANL1000*	0.20±0.01
koANLS-*ANL1000*	0.23±0.03
kocbhS-*ANL1000*	0.32±0.02

^*^The standard deviation indicates three biological replicates of three transformants for each construct.

**Table 3:** The kinetic parameters of ANL.

Lipase	*K*_m_ (g/L)	*k*_cat_(1/s)	*k*_cat_/*K*_m_(L/(s g))
ANL	9.30±1.04	391.66±8.69	42.25±3.35
