# Supplementary material for: Improved Homologous Expression of the Acidic Lipase from Aspergillus niger
Source: J Microbiol Biotechnol. 2019 Nov 22;30(2):196–205. doi: 10.4014/jmb.1906.06028 (PMC9728306; doi:10.4014/jmb.1906.06028)
Supplement: Supplementary file 1 [file JMB-30-2-196-supple.pdf]

Journal of Microbiology and Biotechnology

J. Microbiol. Biotechnol.
https://doi.org/10.4014/jmb.1906.06028

Supplementary

Supplementary Table 1 List of primers used for vector construction

	Primer name	Primer sequence (5'-3')
P1	ANL-F	ATGTTCTCTGGACGGTTTGG
P2	1ANL-R	TCCAACGTGGAAGTCGAGACGCTCCGCACATCAAGTGGT
P3	2ANL-F	CACCACTTGATGTGCGGAGTGTCTCGACTTCCACGTTG
P4	2ANL-R	GTGCCTCCAAAGTTATTTGTCAGGTCAAACTCCAGCAGC
P5	3ANL-F	TGCTGCTGGAGTTCGACCTGACAAATAACTTTGGAGGCACA
P6	3ANL-R	CGAGGACATCCATAGGTGTACAGTTCAACACTATAACCGTC
P7	4ANL-F	GACGGTTATAGCGTTGAACTGTACACCTATGGATGTCCTCG
P8	4ANL-R	TAGCAGACACTCTGAAATTGC
P9	pCAMANL-F	AGT***GGGCCC***GCACCGACACCACTTGATG
P10	pCAMANL-R1	TTAATGATGATGATGATGATGGCTGCTGCCTAGCAGACACTC TGAAATTGCG
P11	pCAMANL-R2	CAG***AGATCTGGATCC***TTAATGATGATGATGATGATGGCT
P12	kogpdA-F	ACT***GGTACC***CGGAGAATATGGAGCTTCATC
P13	kogla-R1	CTCAGGGCGAGTAGAGATCGGAACGAATCCATGGTGGCGGT GATGTCTGCTCAAGCGG
P14	kogla-R2	cat***GGGCCC***TGCCAACCCTGTGCAGACGAGGCCGCTCAGGG CGAGTAGAGATCG
P15	koANL-R1	AAAAGCACTCCAAACCGTCCAGAGAACTCCATGGTGGCGG TGATGTCTGCTCAAGCGG
P16	koANL-R2	cat***GGGCCC***CGCAGCACTCAGCGCAGCGAGCGCCGTCAAAA GCACTCCAAACCGTCCA
P17	kocbh-R1	CCGAGATGACGGCCAACTTCCGATACTCCATGGTGGCGGTG ATGTCTGCTCAAGCGG
P18	kocbh-R2	cat***GGGCCC***CGACTGAGCACGAGCTGTGGCCAAGAAGGCCG AGATGACGGCCAACTTC

* The bold and italic parts are the restriction enzyme sites.

Supplementary Table 2 List of identification primers

	Primer name	Primer sequence (5'-3')
P19	Ident-hyg-F	CTATTTCTTTGCCCTCGGAC
P20	Ident-hyg-R	ATGAAAAAGCCTGAACTCACC
P21	gpdA-F	GCAGGTACCCGGAGAATATGGAGCTTC
P8	4ANL-R	TAGCAGACACTCTGAAATTGC

Supplementary Table 3 List of signal sequences

	Signal name	The signal sequences
1	*glaA* signal	**GCCACC**ATG**G**ATTCGTTCCGATCTCTACTCGCCCTGAGCGGCCTCGTCTGCACAGGGTTGGCA
2	*ANL* signal	**GCCACC**ATG**G**AGTTCTCTGGACGGTTTGGAGTGCTTTTGACGGCGCTCGCTGCGCTGAGTGCTGCG
3	*cbh* Ⅰ signal	**GCCACC**ATG**G**AGTATCGGAAGTTGGCCGTCATCTCGGCCTTCTTGGCCACAGCTCGTGCTCAGTCG

* The bold parts are the kozak sequence. The underlined two nucleotides in each signal sequence were added downstream of the kozak sequence G^{+4} to avoid frameshift.
